# Determinants of participation and detection rate of upper gastrointestinal cancer from population‐based screening program in China

**DOI:** 10.1002/cam4.2578

**Published:** 2019-09-27

**Authors:** Lanwei Guo, Shaokai Zhang, Shuzheng Liu, Liyang Zheng, Qiong Chen, Xiaoqin Cao, Xibin Sun, Youlin Qiao, Jiangong Zhang

**Affiliations:** ^1^ Department of Cancer Epidemiology Henan Office for Cancer Control and Research The Affiliated Cancer Hospital of Zhengzhou University Henan Cancer Hospital Zhengzhou China; ^2^ Office of Cancer Screening National Cancer Center/National Clinical Research Center for Cancer/Cancer Hospital Chinese Academy of Medical Sciences and Peking Union Medical College Beijing China

**Keywords:** adherence, early detection, endoscopy, lesion

## Abstract

Upper gastrointestinal cancer (UGC) screening has been widely implemented in many Asian countries. However, there is little evidence of participation and diagnostic yields in population‐based UGC screening in China. The participation rate and detection of upper gastrointestinal lesions in this program were reported and related factors were explored. The analysis was conducted in the context of the Cancer Screening Program in Urban China, which recruited 179 002 eligible participants aged 40‐74 years from three cities in Henan province from 2013 to 2017. A total of 43 423 participants were evaluated to be high risk for esophageal cancer or gastric cancer by an established risk score system and were subsequently recommended for endoscopy. Of 43 423 with high risk for UGC, 7996 subjects undertook endoscopy (participation rate of 18.4%). We found that male sex, high level of education, marriage, smoking, current alcohol drinking, lack of physical activity, history of upper gastrointestinal system disease, and family history of UGC were associated with increased participation of endoscopy screening. Overall, 15 UGC (0.19%), 275 squamous epithelial dysplasia (3.44%), and 33 intraepithelial neoplasm (0.41%) cases were detected. Several factors including age, sex, smoking, current alcohol drinking, lack of physical activity, and dietary intake of processed meat were identified to be associated with the presence of upper gastrointestinal lesions. Health promotion campaigns targeting the specific group of individuals identified in our study will be helpful for improvement of the adherence of UGC screening in population‐based cancer screening programs. Participant rate and yield of UGC screening will provide important references for evaluating the effectiveness and cost‐effectiveness of cancer screening in China.

## Graphical abstract

Endoscopy is effective in terms of detecting esophageal cancer, gastric cancer, and precursors. The adherence to screening endoscopy in this high‐risk population is relatively low and factors associated with the willingness to accept endoscopy screening were identified. Risk factors associated with upper gastrointestinal lesion in Chinese population were identified.

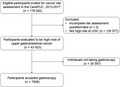

## 1 INTRODUCTION

Upper gastrointestinal cancer (UGC, including esophageal cancer and gastric cancer) is the most commonly diagnosed cancer worldwide, with age‐standardized incidence rate of 17.4 per 100 000 and age‐standardized mortality rate of 13.7 per 100 000, in 2018.^1^ In China, although the incidence and mortality has slightly decreased,^2^ high burden of UGC still persists. The world‐standardized incidence and mortality of UGC in China were 34.6 and 30.2 per 100 000, respectively, which ranked third and second in the world. A total of 763 483 incident cases and 673 615 deaths were estimated in 2018, which accounted for 47.5% and 52.2% of the world cases.^1^

Randomized controlled trials and observations studies have shown that endoscopic screening carry large potential for reducing the burden of esophageal cancer^3^, ^4^ and gastric cancer.^5^, ^6^ To date, organized and opportunistic screening programs have been widely implemented in many Asian countries.^7^, ^8^ However, such screening programs were mainly implemented in high‐income countries which typically have high incidence rates of gastric cancer. Endoscopy with biopsies for histologic confirmation is regarded as the gold standard of UGC screening, but it is an invasive procedure requiring high level of expertise. In countries having intermediate or low incidence rates of UGC and limited healthcare resources, risk‐stratified scoring system was recommended to select high‐risk patients for endoscopy.^9^, ^10^ However, evidence confirming the effectiveness of such strategies combining risk stratification and subsequent endoscopy in population‐based screening programs is still sparse. Since October 2012, the Chinese government initiated a population‐based Cancer Screening Program in Urban China (CanSPUC), which targeted five types of cancer that are most prevalent in urban areas, including lung cancer, female breast cancer, liver cancer, UGC, and colorectal cancer. This program began in Henan in 2013. Eligible participants are recruited in the communities of the study regions and invited to undertake cancer screening free of charge. Participants are first invited to take a cancer risk assessment by an established Clinical Cancer Risk Score System, and those who are evaluated to be high risk for specific types of cancer are recommended to take appropriate screening intervention per study protocol. For UGC screening, participants of high risk for esophageal cancer or gastric cancer are recommended to take subsequent endoscopy at tertiary‐level hospitals designated by the program.

In this study, we report the results of UGC cancer screening conducted in the first 4 years of this cancer screening program in Henan province of China between October 2013 and October 2017. We aim to provide timely evidence of the participation and diagnostic yield of endoscopy screening in high‐risk populations in China and to provide important references for designing effective UGC screening strategies in the future.

## 2 METHODS

### 2.1 Study design and population

A cross‐sectional study was conducted under the framework of CanSPUC. The CanSPUC was launched in October 2012, which is an ongoing national cancer screening program. In short, residents aged 40 to 74 years old, who living in the selected communities of the participating cities, were approached by trained staffs by means of phone calls and personal contact. Social media and community advertisement were used to raise the public awareness of this cancer screening program. All eligible participants were interviewed by trained staff to gather information about their exposure to risk factors and, after giving signed written informed consent, had their cancer risk assessed using a defined risk score system. In order to optimize the use of limited healthcare resources and to increase the detection rate of UGC neoplasia in this screening program, only participants who were assessed to be at high risk of esophageal cancer or gastric cancer were recommended to undergo endoscopy examination at a tertiary‐level hospital that was designated by the program and free of charge. The study was approved by the Ethics Committee of National Cancer Center/Cancer Hospital, Chinese Academy of Medical Sciences and Peking Union Medical College and all participants provided written informed consent.

**Figure 1:**
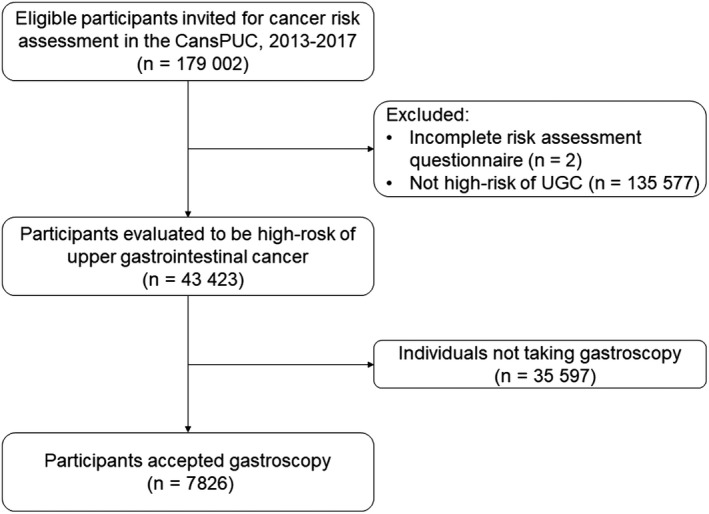
Flow diagram of participant recruitment in CanSPUC, 2013‐2017

For the present analyses, we used the UGC screening data conducted in the first 4 years between October 2013 and October 2017 in Henan province, which covered a total of three cities (Zhengzhou, Zhumadian, and Anyang). Overall, there were 179 002 eligible participants recruited. After excluding participants not at high risk for esophageal cancer or gastric cancer (N = 135 577) and those with invalid risk assessment results (N = 2), the present study included 43 423 remaining participants. A flowchart showing the recruitment of study population is shown in Figure 1.

### 2.2 Risk assessment

Participants were asked to undertake the risk assessment before endoscopy. The rationale of the development of the cancer risk score system is based on the Harvard Risk Index,^11^ but it also included risk factors, relative risks, and exposure rates of risk factors specific to the Chinese population. The risk scoring system includes the following factors: smoking, alcohol drinking, tea drinking, dietary intake of pickled food, hot drink or hot food diet, indoor soot exposure in the past 10 years, history of upper gastrointestinal system diseases, body mass index (BMI), alcohol drinking, high‐salt diet, more‐dry diet, history of trauma, history of chronic gastritis, history of duodenal ulcer, and family history of esophageal or gastric cancer in first‐degree relative. Each risk factor was assigned a score by a panel of experts based on the magnitude of its association with esophageal or gastric cancer.

### 2.3 Clinical procedures

All gastroscopies were conducted in the four tertiary‐level hospitals by experienced gastroenterologists (attending physician or above having experiences of endoscopy for at least 5 years). Abnormal findings during endoscopy were carefully checked under standard clinical procedures (endoscopy with Lugol's iodine staining) and biopsies were collected for further pathology diagnosis. Any findings during endoscopy were photo documented. Clinical information such as morphological feature, macroscopic diagnosis, and size were collected and documented in the data system. Participants who had incomplete endoscopy were asked to retake the endoscopy exam to meet the clinical standard for diagnosis.

### 2.4 Outcome ascertainment and quality control

All abnormal findings discovered during endoscopy were confirmed by the pathological examination following up‐to‐date clinical guidelines. Pathologists were required to complete highly standardized forms to collect pathology results. For difficult cases, consultation by the expert panel from the National Cancer Center of China was conducted and reviewed reports were transferred to the respective physicians about the consultation results.

In this study, squamous epithelial dysplasia included squamous epithelial low‐grade intraepithelial neoplasia, squamous epithelial high‐grade intraepithelial neoplasia, squamous intraepithelial neoplasia that cannot be classified, and suspected infiltration of squamous epithelial high‐grade intraepithelial neoplasia. Intraepithelial neoplasm included glandular epithelial low‐grade intraepithelial neoplasia and glandular epithelial high‐grade intraepithelial neoplasia.

### 2.5 Data acquisition

Trained staff and physicians were responsible for collecting paper‐based standardized documentation forms (epidemiological questionnaire, endoscopy report and pathology report, etc). Validity of forms was checked and entered into the data management system by trained study staff. A consistency check was performed and if an inconsistency was found, the error was corrected by retrieving the original record. Each participant had a unique identifier that was used to track all of the individual's relevant documentation forms. All data were transmitted to the Central Data Management Team in the National Cancer Center of China where the databases were constructed and analyses were performed.

### 2.6 Statistical analysis

In addition to the descriptive analyses regarding the characteristics of the study population, overall and group‐specific participation rates by common factors were calculated and respective 95% confidence interval (95% CI) were reported. Differences in participation rates between different groups were compared using the Chi‐square test. Associations between factors—including age (categorized into 40‐44, 45‐49, 50‐54, 55‐59, 60‐64, 65‐69, and 70‐74 years), sex (male, female), body mass index (<18.5, 18.5‐24.0, 24.0‐28.0 and ≥28.0 kg/m^2^), education background (low: primary school or below, intermediate: junior or senior high school, high: undergraduate or over), marriage status (unmarried or divorce or widowed, married), smoking status (never, current, former), alcohol drinking (never, current, former), physical activity (<3 times/wk, ≥3 times/wk), history of upper gastrointestinal system disease (yes, no), and family history of CRC (yes, no)—and participation rate in endoscopy were quantified by odds ratios (ORs) and their 95% CIs. These were derived by multiple logistic regression models after adjusting for factors including ethnicity, occupation, recruitment, and study sites. Diagnostic yield of screening endoscopy—including esophageal and gastric findings, age‐ and sex‐detection rates, and distribution of location of neoplasms—was calculated. Yield per 10 000 invitees and number of gastroscopies to detect one upper gastrointestinal lesion were also calculated. Associations of various characteristics with prevalence of esophageal and gastric neoplasms were likewise quantified by ORs and their 95% CIs using logistic regression models. All statistical analyses were performed with the statistical software SAS version 9.4 (SAS Institute). All tests were two sided and *P*‐values of .05 or less were considered to be statistically significant.

## 3 RESULTS

### 3.1 Characteristics of the study population

As shown in Figure 1, there were 179 002 eligible participants recruited in the Henan Province in 2013‐2017. After excluding participants with incomplete risk assessment questionnaires (N = 2) and participants whose results of risk assessment were not high risk for esophageal and gastric cancer (N = 135 577), 43 423 remaining participants with high risk of esophageal or gastric cancer were included in the final analyses. Characteristics of the high‐risk population of UGC are presented in Table 1. Overall, more women (56.0%) were included in the study. The mean age was 55.5 years (standard deviation = 8.2 years), and the majority (73.7%) were between 45 and 64 years old. About 54% of the participants (N = 23 410) were overweight or obese, and about 60% of them (N = 25 947) participated in physical activity less than three times a week. About 54% of the participants (N = 16 333) were current smokers or past smokers, and about 43% of them (N = 23 410) are current or past drinker. About 23% of the participants (N = 41 901) had a history of previously detected upper gastrointestinal system disease, and about 35% of them (N = 15 342) had a family member diagnosed with UGC.

**Table 1:** Characteristic of the study population and participation rates between different groups

Factors	Participants of high risk for UGC (%)	Participants undertaking gastroscopy (%)	Participation rate (%)	χ^2^	*P‐*value
Age (y)				86.01	<.001
40‐44	4358 (10.04)	768 (9.60)	17.62		
45‐49	7616 (17.54)	1462 (18.28)	19.20		
50‐54	8824 (20.32)	1725 (21.57)	19.55		
55‐59	7378 (16.99)	1422 (17.78)	19.27		
60‐64	8199 (18.88)	1545 (19.32)	18.84		
65‐69	5524 (12.72)	904 (11.31)	16.36		
70‐74	1524 (3.51)	170 (2.13)	11.15		
Sex				4.93	.026
Male	19 105 (44)	3429 (42.88)	17.95		
Female	24 318 (56)	4567 (57.12)	18.78		
BMI (kg/m^2^)				3.42	.331
<18.5	990 (2.28)	175 (2.19)	17.68		
18.5‐24.0	19 023 (43.81)	3547 (44.36)	18.65		
24.0‐28.0	18 213 (41.94)	3360 (42.02)	18.45		
≥28.0	5197 (11.97)	914 (11.43)	17.59		
Education background				72.79	<.001
Primary school or below	6214 (14.31)	991 (12.39)	15.95		
Junior/Senior high school	28 953 (66.68)	5246 (65.61)	18.12		
Undergraduate or over	8256 (19.01)	1759 (22.00)	21.31		
Marriage				13.03	<.001
Unmarried/Divorce/Widowed	2119 (4.88)	453 (5.67)	21.38		
Married	41 304 (95.12)	7543 (94.33)	18.26		
Smoking				12.13	.002
Never	27 090 (62.39)	4864 (60.83)	17.95		
Current	13 228 (30.46)	2509 (31.38)	18.97		
Former	3105 (7.15)	623 (7.79)	20.06		
Alcohol drinking				24.73	<.001
Never	24 701 (56.88)	4364 (54.58)	17.67		
Current	16 517 (38.04)	3236 (40.47)	19.59		
Former	2205 (5.08)	396 (4.95)	17.96		
Physical activity				79.91	<.001
<3 times/wk	25 947 (59.75)	5132 (64.18)	19.78		
≥3 times/wk	17 476 (40.25)	2864 (35.82)	16.39		
History of upper gastrointestinal system disease				172.92	<.001
No	8259 (19.02)	1104 (13.81)	13.37		
Yes	35 164 (80.98)	6892 (86.19)	19.60		
Family history of UGC					
No	28 081 (64.67)	4712 (58.93)	16.78		
Yes	15 342 (35.33)	3284 (41.07)	21.41		

Abbreviations: BMI, body mass index; UGC, upper gastrointestinal cancer.

### 3.2 Participation rate of screening endoscopy and its associated factors

Of the 43,423 participants of high risk for UGC, 7,996 of them undertook endoscopy as recommended by our study. The overall participation rate was 18.4% (95% CI: 18.1%‐18.7%). The participation rates stratified by potential associated factors are shown in Table 1. Overall, the participation rates were slightly higher in women than in men (18.8% vs 18.0%, *P*‐value = .026). They were also higher among participants aged 45‐64 years. Univariate analyses showed that participants who had a high educational background, were unmarried/divorce/widowed, were current or past smokers, were current alcohol drinkers, lacked physical activity, had a history of upper gastrointestinal system disease, or had a family history of UGC had relatively higher participation rates.

As shown in Table 2, we also conducted multivariable logistic regression models to explore the potential factors that were associated with PR. After adjusting for ethnicity, occupation, and BMI, we found that age, sex, education background, marriage, smoking, alcohol drinking, physical activity, history of upper gastrointestinal system disease, and family history of UGC were associated with participation rate. For instance, the odds of a participant with a history of upper gastrointestinal system disease undertaking screening endoscopy was 0.9‐fold higher than for a participant with no history of upper gastrointestinal system disease (OR: 1.89, 95% CI: 1.76%‐2.03%). Similarly, the odds of a participant with a family history of UGC undertaking screening endoscopy was 0.6‐fold higher than for a participant with no family history of UGC (OR: 1.56, 95% CI: 1.48%‐1.65%). Since participation rate varied between study site and year of participant recruitment, these two factors were additionally adjusted in the model II and the respective ORs did not change greatly (see Table 2).

**Table 2:** Odds ratios of factors associated with participation rate of gastroscopy in the screening program

Factors	Model I^a^		Model II^b^	
	Odds ratio (95% CI)	*P*‐value	Odds ratio (95% CI)	*P*‐value
Age (y)				
40‐44	1.00			
45‐49	1.14 (1.03‐1.25)	.010	1.15 (1.04‐1.27)	.005
50‐54	1.18 (1.07‐1.29)	.001	1.19 (1.08‐1.31)	<.001
55‐59	1.19 (1.08‐1.32)	.001	1.20 (1.08‐1.33)	<.001
60‐64	1.18 (1.07‐1.30)	.001	1.20 (1.08‐1.32)	<.001
65‐69	1.03 (0.92‐1.15)	.578	1.05 (0.94‐1.17)	.387
70‐74	0.67 (0.56‐0.80)	<.001	0.75 (0.63‐0.90)	.002
Sex				
Male	1.00			
Female	1.23 (1.14‐1.32)	<.001	1.24 (1.15‐1.34)	<.001
Education background				
Primary school or below	1.00			
Junior/Senior high school	1.16 (1.07‐1.26)	<.001	1.17 (1.08‐1.27)	<.001
Undergraduate or over	1.36 (1.22‐1.51)	<.001	1.36 (1.23‐1.51)	<.001
Marriage				
Unmarried/Divorce/Widowed	1.00			
Married	1.25 (1.12‐1.40)	<.001	1.28 (1.15‐1.43)	<.001
Smoking				
Never	1.00			
Current	1.09 (1.01‐1.18)	.036	1.11 (1.02‐1.20)	.016
Former	1.24 (1.11‐1.38)	<.001	1.24 (1.11‐1.39)	<.001
Alcohol drinking				
Never	1.00			
Current	1.15 (1.08‐1.23)	<.001	1.15 (1.07‐1.23)	<.001
Former	0.98 (0.87‐1.11)	.737	0.97 (0.86‐1.10)	.673
Physical activity				
<3 times/wk	1.20 (1.14‐1.27)	<.001	1.22 (1.16‐1.29)	<.001
≥3 times/wk	1.00			
History of upper gastrointestinal system disease				
No	1.00			
Yes	1.89 (1.76‐2.03)	<.001	1.88 (1.74‐2.02)	<.001
Family history of UGC				
No	1.00			
Yes	1.56 (1.48‐1.65)	<.001	1.60 (1.51‐1.68)	<.001

a Odds ratios were adjusted for factors including ethnicity, occupation, and BMI (<18.5 kg/m^2^, 18.5‐24.0 kg/m^2^, 24.0‐28.0 kg/m^2^, ≥28.0 kg/m^2^) in the logistic regression model.

b Except for the factors included in the model I, odds ratios were additional adjusted for year of recruitment and study sites in logistic regression model.

### 3.3 Upper gastrointestinal findings under screening endoscopy

Table 3 presents the diagnostic yield of endoscopy in our screening program. Overall, there were 15 UGC, 275 squamous epithelial dysplasia, and 33 intraepithelial neoplasm cases. This yielded the detection rates for UGC, squamous epithelial dysplasia, and intraepithelial neoplasm at 0.19%, 3.44%, and 0.41%, respectively. Furthermore, based on the sex‐ and age‐adjusted detection rates for the standard population of China (1982), we calculated that the number of gastroscopies needed to detect one esophageal or gastric cancer, one squamous epithelial dysplasia, and one intraepithelial neoplasm as 1086, 63, and 417, respectively. In terms of diagnostic yield per invitees, 3 UGC, 344 squamous epithelial dysplasia, and 42 intraepithelial neoplasm cases could be detected per 10 000 invitees.

**Table 3:** Esophageal and gastric lesions detected by gastroscopy in the screening program

Findings	Number detected and detection rate (%)	Yield per 10,000 invitees	Number of gastroscopy to detect one lesion^a^
UGC	15 (0.19)	3	1086
Esophageal cancer	8 (0.10)	2	1602
Gastric cancer	7 (0.09)	2	3396
Squamous epithelial dysplasia	275 (3.44)	344	63
Intraepithelial neoplasm	33 (0.41)	42	417

a Calculation was based on the age‐ and sex‐specific detection rate adjusted by China Standard Population (1982).

**Figure 2:**
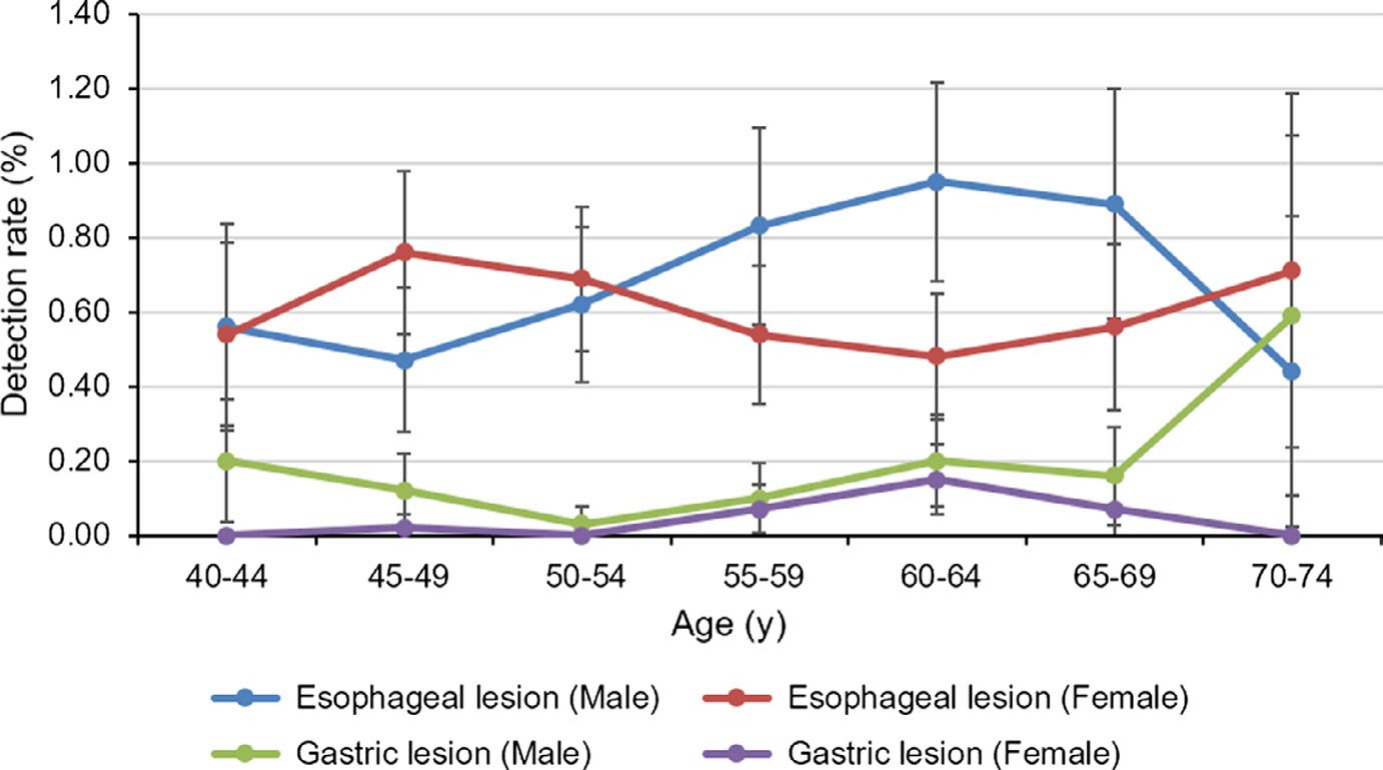
Detection rates of esophageal lesion and gastric lesion stratified by age and sex

The detection rates for esophageal and gastric lesions increased with age and were higher for men than for women, as shown in Figure 2. In particular, the detection rate of esophageal lesions for men aged 60‐64 years was 0.95% (95% CI: 0.68%‐1.22%), significantly higher than respective rate for women at the same age range (detection rate: 0.48%; 95% CI: 0.31%‐0.65%).

### 3.4 Factors associated with the neoplasms detection of screening endoscopy

**Figure 3:**
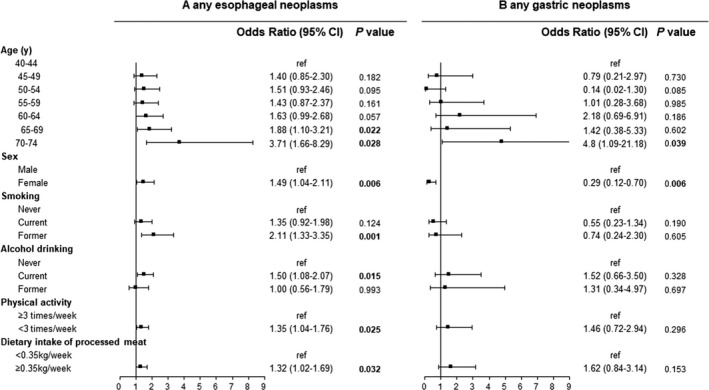
Odds ratio of risk factors associated with any esophageal neoplasms and gastric neoplasms. Analyses were adjusted for age, sex, BMI, family history of upper gastrointestinal cancer (UGC), history of upper gastrointestinal system disease, dietary intake of coarse grains, fresh vegetables, processed meat, smoking, alcohol drinking, physical activity, ethnicity, education background, year of recruitment, and study site

We further performed multivariate logistic regression analyses to identify potential risk factors that may be associated with upper gastrointestinal neoplasms in the Chinese population. These results are shown in Figure 3. Older age, female sex, previous smoking, regular drinking, lack of physical activity, and high levels of dietary intake of processed meat had a positive association with esophageal neoplasms. Older age and male sex had a positive association with gastric neoplasms. For instance, when comparing against individuals aged 40‐44 years, the ORs (95% CI) of individuals aged 45‐49, 50‐54, 55‐59, 60‐64, 65‐69, and 70‐74 years carrying esophageal neoplasms were 1.40 (0.85‐2.30), 1.51 (0.93‐2.46), 1.43 (0.87‐2.37), 1.63 (0.99‐2.68), 1.88 (1.1‐3.21), and 3.71 (1.66‐8.29), respectively. Former smoking, regular drinking, lack of physical activity, and high levels of dietary intake of processed meat were only positively associated with esophageal neoplasms. Other factors including history of upper gastrointestinal system disease, family history of UGC, dietary intake of coarse grain food, and dietary intake of fresh vegetables were found to have no association with the presence of esophageal and gastric lesions in our study population.

## 4 DISCUSSION

We reported the results of 43 423 participants undertaking UGC screening in a population‐based organized cancer screening program in China. To our knowledge, our study is the first to present the participation and diagnostic yield of UGC screening using a strategy combining risk score stratification and endoscopy based on results from a large‐scale cancer screening program in China.

Endoscopy is the gold standard for UGC screening that allows for the detection and removal of early lesion. In Korea, gastric cancer screening by upper gastrointestinal endoscopy and upper gastrointestinal series using ingested barium has been provided for the population aged 40 years and over since 2002.^7^ In 2017, the Japanese Society of Gastrointestinal Cancer Screening approved endoscopic examination for population‐based gastric cancer screening and recommended that the screening should be performed every 2 years in all individuals.^12^ However, the acceptance of endoscopy in general population is not optimal and is still a major challenge in population‐based UGC screening, especially for screening programs using endoscopy as the main screening element. For instance, the participation rate for endoscopic screening in Korea changed from 11.4% in 2002 to 29.2% in 2008.^7^ In Japan, this has been partly adopted in population‐based screening, with a participation rate of 16%.^12^ Through a randomized controlled trial, Wei et al^3^ demonstrated for the first time in the world that endoscopic screening in high‐risk areas of esophageal cancer can reduce the incidence and mortality of esophageal cancer. However, the 10‐year screening effect of this study was obtained on the premise that the population compliance reached 50%. If the participation rate with screening is too low, it will not only waste resources but also have no effect on reduction of morbidity and mortality.

This is the first study to present the participation and diagnostic yield of UGC screening using a strategy combining risk score stratification and endoscopy based on results from a large‐scale cancer screening program in China. In the CanSPUC, the overall participation rates of UGC cancer was slightly higher than that of colorectal cancer screening by colonoscopy (14.0%).^13^ The study suggested that factors such as high level of education, history of fecal occult blood test, family history of colorectal cancer, and history of colonic polyp were key factors for successful colorectal cancer screening uptake.^13^ In our study, factors such as male sex, high level of education, married, smoking, current alcohol drinking, lack of physical activity, history of upper gastrointestinal system disease, and family history of UGC were found to be associated with increased participation of endoscopy screening. The underlying reasons for this increase likely included the invasive nature of endoscopy and poor awareness and knowledge about UGC screening. However, factors that were associated with the nonparticipants were not evaluated in our study and need to be further explored. Our results imply that public awareness campaigns would be necessary to improve the participation rate of UGC screening in the future. Therefore, in the case of low screening participation rates and low health awareness, we believe that even if cancer screening is included in healthcare, the intervention effect is minimal.

The detection rates for esophageal and gastric lesions increased with age and were higher for men than for women, which is consistent with the prevalence of esophageal and gastric cancer in Henan Province.^14^ The fluctuations in the detection rate of each age group may be related to the small number of screening cases and the different participation rates in each age group. We will increase the sample size for verification in future studies. The overall dysplasia detection rate (any grade of dysplasia or early esophageal cancer) in our study population was 3.54%. This was higher than that of some population‐based screening programs in China, but lower than that of some studies conducted in high‐risk areas in rural China and Northern Iran. For instance, in a screening program that had 12 454 participants undertaking screening endoscopy in high‐risk areas in rural China, the overall dysplasia detection rate was 1.99%.^15^ In a cross‐sectional study with 724 participants that undertook screening endoscopy in high‐risk areas in rural China, the overall dysplasia detection rate was 32%.^16^ In another cross‐sectional study that used 302 endoscopy screening records from high‐risk areas in northern Iran, the overall dysplasia detection rate was 9.0%.^17^ The overall intraepithelial neoplasm detection rate (any grade of intraepithelial neoplasm or early gastric cancer) in our study population was 0.50%, which was lower than some studies conducted in high‐risk areas in rural China and other Asian countries. For instance, in a screening program with 12,454 participants that undertook screening endoscopy in the high‐risk areas in rural China, the overall intraepithelial neoplasm detection rate was 0.67%.^15^ In another population‐based screening program that used 924,822 endoscopy screening records from Korea, the overall intraepithelial neoplasm detection rate was 4.2%.^18^ It should be noted that a target rate was defined using evidence mostly from the high‐risk regions which typically have higher prevalence of esophageal and gastric cancer. Besides, the high detection rate is accompanied by a relatively small sample and higher participation rate. Therefore, the low detection rate in our study might be explained by the low participation rate and relatively lower prevalence of esophageal or gastric cancer in urban Henan than in other rural Chinese areas or in other Asian countries.

Our study shows that several sociodemographic factors including age, sex, smoking, alcohol drinking, lack of physical activity, and dietary intake of processed meat have a positive association with esophageal or gastric neoplasms. The associations of these factors with esophageal or gastric neoplasms have been extensively explored and our findings were in line with previous studies.^16^, ^19^, ^20^, ^21^ We noticed that being female was identified to be positively associated with esophageal neoplasms in multivariate logistic regression analyses. We reexamined the results of the univariate analysis and attempted to change the study endpoint to esophageal cancer for reanalysis. None of the above analyses found a correlation between gender and esophageal neoplasms. In our study, we noticed that there were more female participants, whose compliance was much higher than males, and whose detection rate was higher than in males in some age groups. All of the above reasons may lead to a higher risk for esophageal neoplasms in females in multivariate analysis. Because certain factors, such as smoking, were also included in the risk score system to select high‐risk populations, the degree of association may be underestimated. However, these analyses are essential for validating precontained factors and exploring new risk factors, with the goal of further optimizing risk assessment models for future research.

It deserves to be noted that in our study we found that the overall detection rate for esophageal or gastric neoplasms was low in a high‐risk population in urban China. Given the relatively low participation rate of screening endoscopy, detection rates were even lower when calculated per invitees. To further improve the screening yield of UGC screening in China, the following issues need to be addressed in next‐step researches: (a) to optimize the risk assessment score based on the current study findings and other well‐established risk prediction scores^22^, ^23^, ^24^, ^25^; (b) to explore the role of noninvasive tests as supplement to screening endoscopy; (c) to design novel risk‐adapted screening strategies coving both high‐risk and low‐risk populations using appropriate screening modalities; and (d) to carry out multifactor interventions targeting multiple levels of care with the purpose of optimizing UGC screening acceptance.

Specific strengths and limitations deserve careful attention when interpreting our results. One of the main strengths of our research is that our analyses were the first to illustrate the participation and diagnostic yield of screening endoscopy in a large‐scale population‐based cancer screening program in China. In addition, detailed patient information was collected in a standardized manner by trained study staff, including epidemiological questionnaire and clinical examination data (endoscopy and pathology examination), to ensure the data quality. A team performed competence training and a central review of endoscopy and pathology reports each year to improve the consistency and accuracy of clinical diagnosis. However, there were several limitations in this study. Though our study population was selected from four cities, our study is not representative of the entire general population of Henan province and therefore selection bias cannot be ruled out. Secondly, given that follow‐up work for patients diagnosed with esophageal gastric cancer is still underway, clinical disease information has not been fully obtained. Therefore, tumor stage information was not reported in our study.

In summary, in this large‐scale UGC screening program in China, we found that there was room for improvement regarding UGC screening yield given the relatively low participation rate. We further identified several factors associated with the participation rate of screening endoscopy and several risk factors of esophageal or gastric neoplasms. Our findings will provide important references for designing effective population‐based UGC screening strategies in the future.

## CONFLICT OF INTEREST

All authors disclose no conflict of interest.

## AUTHOR CONTRIBUTIONS

LG, XS, and JZ were involved in conception and design. LG and LZ were involved in statistical analyses. LG, SZ, SL, LZ, QC, XC, and YQ were involved in data acquisition and data interpretation. LG were involved in drafting of the article. All authors revised the manuscript and approved the final version of the manuscript.

## Data Availability

The datasets for this manuscript are not publicly available because all our data are under regulation of both the National Cancer Center of China and Henan Cancer Hospital. Requests to access the datasets should be directed to Jiangong Zhang, jgzhang01@126.com.
